# *ppk23*-Dependent Chemosensory Functions Contribute to Courtship Behavior in *Drosophila melanogaster*

**DOI:** 10.1371/journal.pgen.1002587

**Published:** 2012-03-15

**Authors:** Beika Lu, Angela LaMora, Yishan Sun, Michael J. Welsh, Yehuda Ben-Shahar

**Affiliations:** 1 Department of Biology, Washington University, St. Louis, Missouri, United States of America; 2 Neuroscience Graduate Program, Roy J. and Lucille A. Carver College of Medicine, University of Iowa, Iowa City, Iowa, United States of America; 3 Howard Hughes Medical Institute, Departments of Internal Medicine, Molecular Physiology, and Biophysics, Roy J. and Lucille A. Carver College of Medicine, University of Iowa, Iowa City, Iowa, United States of America; Stanford University School of Medicine, United States of America

## Abstract

Insects utilize diverse families of ion channels to respond to environmental cues and control mating, feeding, and the response to threats. Although degenerin/epithelial sodium channels (DEG/ENaC) represent one of the largest families of ion channels in *Drosophila melanogaster*, the physiological functions of these proteins are still poorly understood. We found that the DEG/ENaC channel *ppk23* is expressed in a subpopulation of sexually dimorphic gustatory-like chemosensory bristles that are distinct from those expressing feeding-related gustatory receptors. Disrupting *ppk23* or inhibiting activity of *ppk23*-expressing neurons did not alter gustatory responses. Instead, blocking *ppk23*-positive neurons or mutating the *ppk23* gene delayed the initiation and reduced the intensity of male courtship. Furthermore, mutations in *ppk23* altered the behavioral response of males to the female-specific aphrodisiac pheromone 7(Z), 11(Z)-Heptacosadiene. Together, these data indicate that *ppk23* and the cells expressing it play an important role in the peripheral sensory system that determines sexual behavior in *Drosophila*.

## Author Summary

Insects utilize diverse families of ion channels to respond to environmental cues and control mating, feeding, and the response to threats. Degenerin/epithelial sodium channels (DEG/ENaC) represent one of the largest families of ion channels in *Drosophila melanogaster*. However, the physiological functions of the majority of these proteins in *Drosophila* or any other animal are still unknown. We discovered that the DEG/ENaC channel *ppk23* is expressed in a subpopulation of sexually dimorphic gustatory-like chemosensory neurons. We show that *ppk23* and the neurons expressing it are important for normal male sexual behaviors, possibly via the detection of an aphrodisiac pheromone, but not for feeding decisions. Together, these data indicate that DEG/ENaC signaling plays an important role in the peripheral sensory system that determines sexual behavior in *Drosophila*.

## Introduction

Animals have evolved a variety of mechanisms to monitor their chemical environment and to guide their behavior, many of which involve ion channels. For example, in vertebrates and insects, several members of the Transient Receptor Potential (TRP) channel family act as chemosensors for noxious stimuli [1]. Furthermore, insect olfactory receptors likely function as ligand-gated ion channels [2], [3]. Recently, a novel family of variant glutamate ionotropic receptors was also identified to contribute to olfactory functions in *Drosophila melanogaster* [4]. This diversity of ion channels suggested that additional ion channels might also play important chemosensory roles.

Additional ion channels that could potentially contribute to chemosensory-driven behaviors are the degenerin/epithelial Na^+^ channels (DEG/ENaC) [5]. DEG/ENaC genes are animal specific and various subunits are expressed in both the peripheral and central nervous systems of invertebrates and vertebrates. DEG/ENaC channels are activated by diverse ligands including protons (acid-sensing ion channels) [5] and the peptide FMRFamide in *Helix aspersa* (FaNaCh channels) [6]. They also contribute to salt sensation [7], [8], mechanosensation [9], and nociception [10]. However, the ligands and physiological function of the majority of DEG/ENaC family members remain unknown.

The *Drosophila* genome encodes 31 DEG/ENaC proteins, called *Pickpocket* (*ppk*) channels [7]. This is a greater number of DEG/ENaC genes than for any other currently sequenced animal genome, including other insects such as the honey bee, which encodes only five DEG/ENaC subunits [11]. The observations that some DEG/ENaC channels function as receptors for extra-cellular signals and that some are expressed in chemosensory organs in *Drosophila* and other species [7], led us to hypothesize that multiple members of the DEG/ENaC family might contribute to chemosensory–driven behaviors in insects.

To test this hypothesis, we used a genetic approach and expression analyses to discover chemosensory specific DEG/ENaC subunits and their possible contributions to behaviors. We have identified *pickpocket 23* (*ppk23*), a member of the DEG/ENaC family, as a gene that is enriched in gustatory sensory neurons that are part of the sex circuit in flies. We demonstrate that *ppk23* and the cells expressing it play a role in normal male-female courtship behavior but not feeding.

## Results

### Identifying potential chemosensory DEG/ENaC channel transcripts

**Figure 1:**
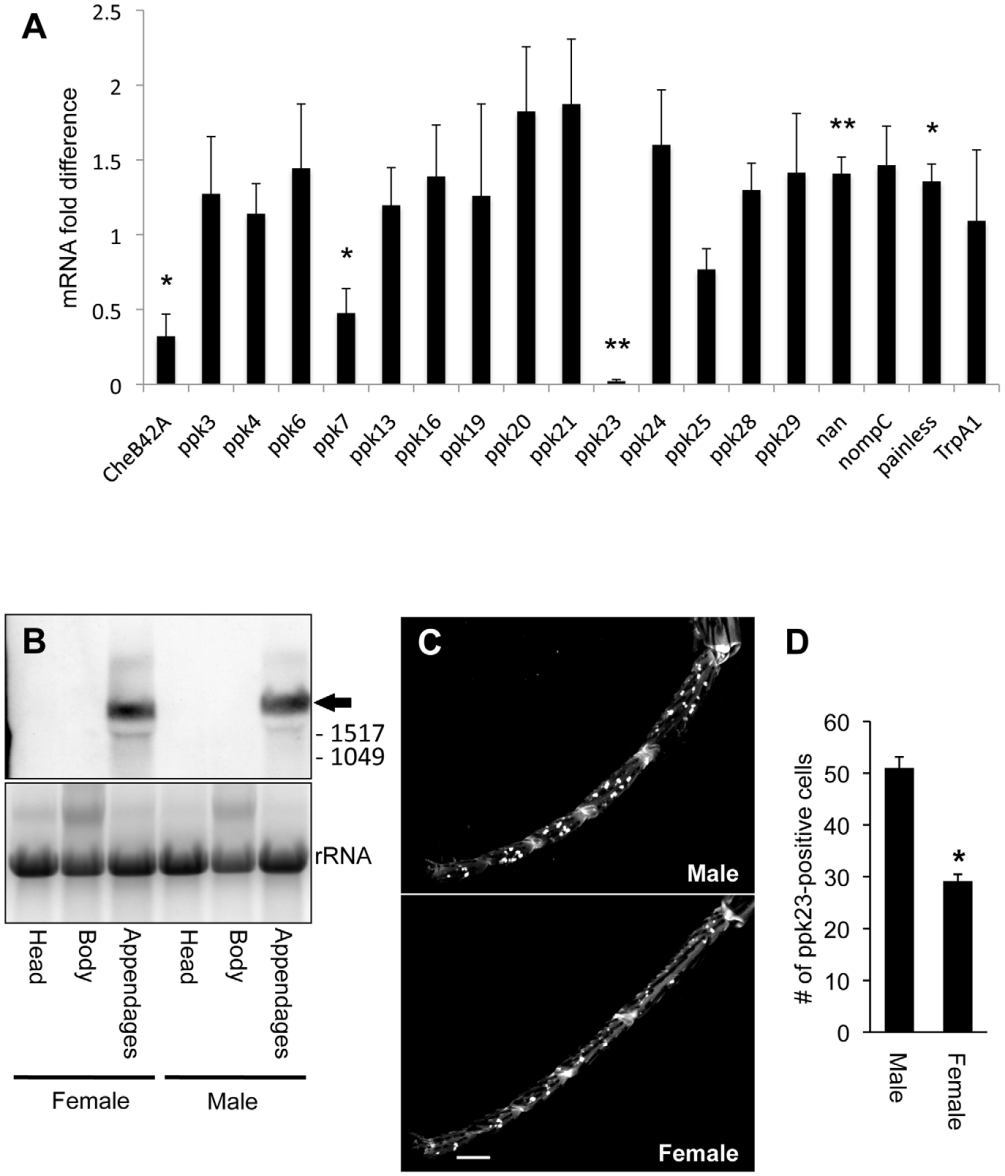
*ppk23* expression is reduced in appendages of the *Poxn* mutant. (A) Real-time quantitative RT-PCR analysis of total RNA extracted from adult appendages (legs and wings) from a mixed sex population. Analysis compared *Poxn* ^M22-B5^, which do not have external chemosensory bristles, and *CyO* balanced siblings, which develop normal sensory bristles [54]. For illustrative purposes, data are represented as relative expression fold differences in *Poxn* flies relative to controls [51]. Each data point includes the relative expression of a gene in *Poxn* homozygous flies relative to balanced *Poxn/CyO* flies, which develop normal sensory system. *CheB42a* gene was used as positive controls for chemosensory specific genes expressed in appendages [16], [17], [55]. Statistical analyses were performed on the ΔCt data as previously described [16], [17], [51]. *, *p*<0.05; **, *p*<0.01 (n = 4 per genotype, one-tail paired *t*-test) (B) Northern blot analysis of *ppk23* spatial expression patterns. Lower panel shows ribosomal bands on the RNA gel, indicating equal sample loading. Using RT-PCR coupled with 5′ and 3′ RACE analyses on RNA extracted from male appendages, we were able to identify only a single *ppk23* transcript (black arrow; *ppk23*-RX; see also Figure S1A). Numbers on right represent RNA size markers (bp). (C) *ppk23* promoter activity in forelegs of male and female flies (reporter was nuclear GFP). Scale bar represents 50 µm. (D) Quantitative data from (C) showing significantly fewer *ppk23*-positive cells in the female foreleg (Independent sample *t*-test; *, p<0.05; n = 10 for each bar).

The *Drosophila melanogaster Pox neuro* (*Poxn*) mutation causes a developmental fate switch of adult chemosensory bristles into pure mechanosensory bristles [12], [13], [14]. We reasoned that comparing gene expression in the sensory-rich appendages of wild-type and *Poxn* adults would reveal novel genes involved in chemosensation. We primarily focused on members of the DEG/ENaC family, which in flies represents one of the largest ion channel families in the fly genome [7], [15]. We used real-time quantitative RT-PCR assays to determine whether the *Poxn* mutation altered the abundance of several DEG/ENaC and sensory TRP channel transcripts. We also examined the chemosensory-related gene *CheB42a* as a positive control (Figure 1A). Compared to the wild type, expression of *ppk23* and *ppk7* were markedly reduced in *Poxn* appendages, suggesting that the function of these genes is associated with chemosensory bristles. Several other DEG/ENaC subunits and several TRP channels showed a mildly increased expression in *Poxn* appendages suggesting that they may contribute to mechanosensation. *lounge lizard* (*llz*, also called *ppk25*), which has been shown to be associated with chemosensory functions [16], [17], [18], showed a very mild reduction in expression that was not statistically significant, likely due to the small sample size used in our screen. In contrast, the dramatic reduction in *ppk23* expression in the *Poxn* genetic background, more than any of the other DEG/ENaC subunits we have looked at thus far, led to our current focus on the role of this specific channel in chemosensory functions. Consistently, northern blot analyses designed to detect all predicted *ppk23* transcripts (See Figure S1A for probe design) showed that *ppk23* expression was highly enriched in appendages (legs and wings) relative to heads or bodies of both males and females (Figure 1B). Real-time quantitative RT-PCR assays also confirmed these results (Figure S1B).

The *ppk23* locus is on the X chromosome and predicted to produce three different transcripts from a single transcriptional start site (FlyBase.org; Figure S1A). A northern blot analysis identified only a single major transcript (Figure 1B). RT-PCR coupled with 5′ and 3′ RACE protocols identified this major transcript as a novel *ppk2*3 transcript (*ppk23*-RX; NCBI accession number HM026485), (Figure S1A). The northern blot analysis also identified two minor bands. We currently do not know their identity, and whether they represent additional minor *ppk23* transcripts or whether this is a non-specific signal. We also have identified an insertional hypomorphic allele of *ppk23*, which significantly reduced the expression of *ppk23* in male appendages (Figure S1C).

To assess the spatial distribution of *ppk23* expression, we used the *ppk23* gene promoter linked to *Gal4* to drive a *UAS-nuclear GFP reporter* in transgenic flies [19]. We found *ppk23* expression enriched in adult appendages (Figure 1C, Figure S1D). When we expressed two copies of *UAS-mCD8-GFP* by two copies of *ppk23 Gal4*, a faint signal in few cells in the labellum was detected as well (data not shown), but not in any other sensory structures. We observed similar expression patterns of *ppk23* in chemosensory neurons in three independent insertions of the same transgenic construct (data not shown). Expression of a nuclear GFP reporter in legs using the *ppk23*-GAL4 strain indicated that although the promoter was active in all legs (Figure S1D), expression in the forelegs of males showed significantly more *ppk23*-positive cells than females (Figure 1C and 1D). Since male forelegs play an important sensory role during courtship [20], these data suggested that the *ppk23* locus might be playing a role in sexual behaviors in flies.

### *ppk23* is enriched in gustatory-like neurons

**Figure 2:**
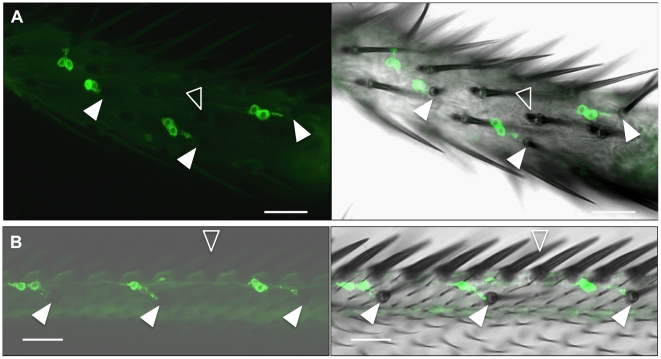
*ppk23* is expressed in adult chemosensory receptor neurons. (A) *ppk23-GAL4 x UAS-CD8::GFP* flies show expression in chemosensory neurons in male forelegs. Left panel is a z-stack of confocal GFP images. Right panel is a merge of the left panel with differential interference contrast (DIC) image. Solid arrows point to the base of bract-less chemosensory bristles. Note the sensory cilia projecting to the base of the bristle. Open arrowhead points to a bract associated with a mechanosensory bristle. Scale bar represent 20 µm. (B) *ppk23-GAL4 x UAS-CD8::GFP* flies show expression in sensory neurons projecting to chemosensory bristles in wings. Images are as in (A) and were obtained from the same animal. Solid arrowheads point to the base of chemosensory bristles. The open arrowhead points to the line of pure mechanosensory bristles found at the most outer rim of the wing. Scale bar represents 20 µm.

Higher resolution analyses of membrane-tethered GFP expression in the male foreleg and wing suggested that *ppk23*-positive cells are chemosensory neurons as evidenced by the projection of their cilium to the base of chemosensory bristles, which were identified by the lack of a bract and a thin, curved morphology [13], [21] (Figure 2A and 2B). We also found that the *ppk23* promoter is active in pairs of sensory neurons with similar morphology that projected to single bristles (Figure 2A and 2B). Because chemosensory bristles contain at most one pure mechanosensitive neuron with distinct morphology [22], this further suggested that *ppk23*-positive neurons are chemosensory.

### Projections of *ppk23*-positive neurons in the foreleg are sexually dimorphic and overlap with *fru*

The chemosensory system in flies plays an important role in regulating social behaviors such as courtship [23]. In addition, recent work indicated that at least some gustatory receptor neurons in fly appendages express the sex-determination gene *fruitless* (*fru*) [24], [25], and have sexually dimorphic axonal projection patterns to the CNS [26]. These data, in combination with the enrichment of *ppk23*-positive cells in the forelegs of males relative to females, led us to hypothesize that *ppk23* and the cells expressing it might contribute to sex-related behaviors.

**Figure 3:**
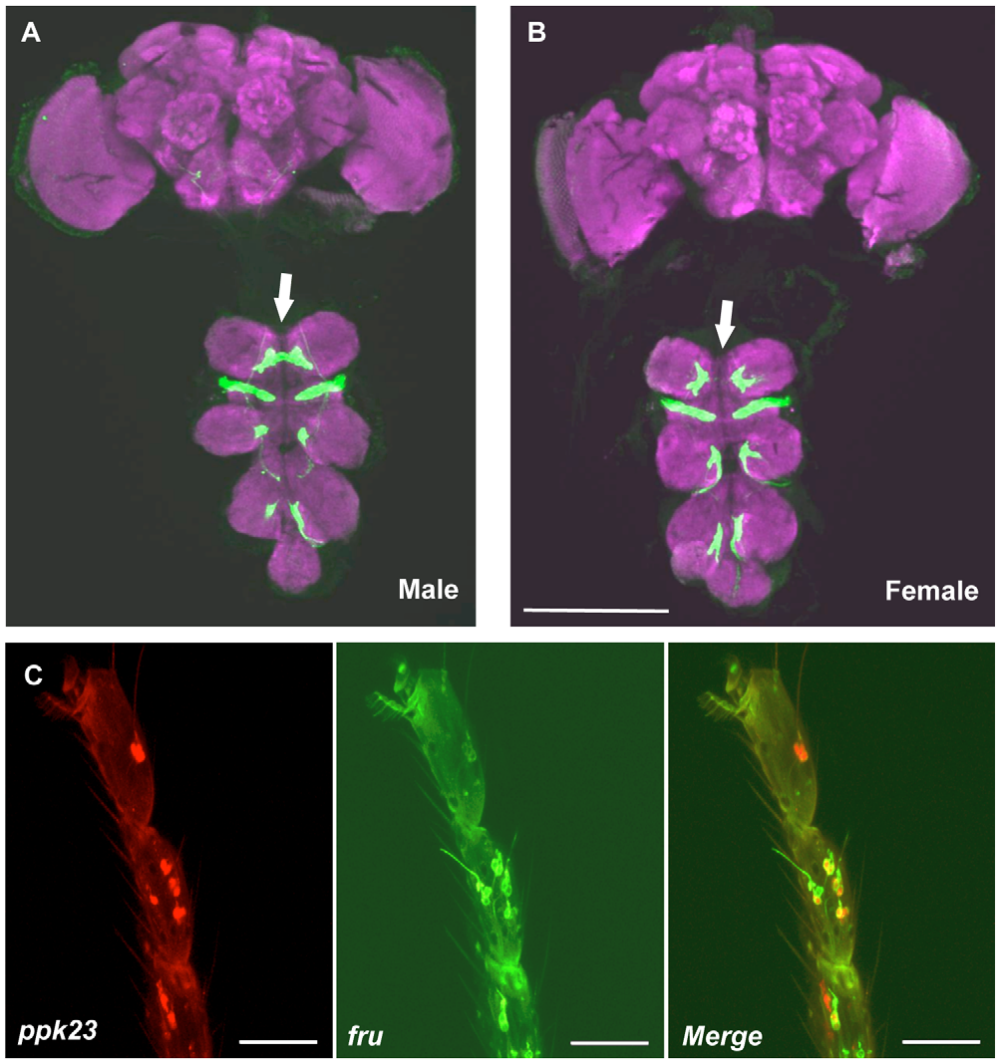
Axonal projections of *ppk23*-expressing cells are sexually dimorphic. (A) and (B) The axonal projections of *ppk23*-positive cells are sexually dimorphic. Left panel is a male (A) and right panel is a female (B). Panels represent brain (top) and thoracic ganglion (bottom) confocal z-stacks that were dissected from a single animal. Note the lack of midline crossings by foreleg *ppk23*-positive axons in females (white arrows). Scale bars represent 100 µm. Identical dimorphic axonal projection patterns were observed in at least five individuals per sex. (C) *ppk23* expression co-localizes with *fru* expression in the forelegs of males. Red marked *ppk23*-expressing cells and green marked the *fru* positive cells. The majority of *ppk23*-expressing cells also show *fru* expression. Scale bars represent 20 µm. Genotype imaged: *w; ppk23-GAL4>UAS-RFP/lexAop-rCD2::GFP; fruP1.LexA/+*.

We first studied the axonal projection patterns of *ppk23*-positive neurons by expressing membrane-tethered GFP using a *ppk23*-GAL4 line and examined GFP patterns in the brain and the thoracic ganglion (Figure 3A and 3B). We did not observe *ppk23*-positive cell bodies in the brains or the thoracic ganglia of either males or females as evidenced by the lack of positive neuronal cell bodies. In contrast, we were able to clearly observe the axonal projection patterns of *ppk23*-positive sensory neurons in the thoracic ganglion as well as weak signal in the subesophageal ganglion. We could not resolve whether these axons represent a small population of labellar sensory neurons or extension of axons that originated in appendages. The axonal projection patterns of *ppk23*-positive sensory neurons from forelegs to the thoracic ganglion were sexually dimorphic, similar to the projections of male specific *Poxn*-positive gustatory neurons, which require *fru^M^* expression for their correct wiring (Figure 3C) [26]; *ppk23*-positive neurons originating in the male foreleg showed higher incidence of crossing the thoracic ganglion midline relative to females (Figure 3A and 3B respectively). The physiological significance of the midline crossing is still unknown.

The possible overlap of *ppk23* expression with the sex circuit was further supported by the co-localization of the *ppk23* promoter with the *fru^P1^* promoter, which is exclusively expressed in the sex circuit (Figure 3C) [24], [25], [26], [27], [28], [29]. Together, these data suggested that the gustatory sensory system includes a subpopulation of sensory neurons in forelegs that express both *fru* and *ppk23* and is probably not involved in feeding-related taste functions [30].

### *ppk23*-expressing cells play a role in normal male courtship behavior

**Figure 4:**
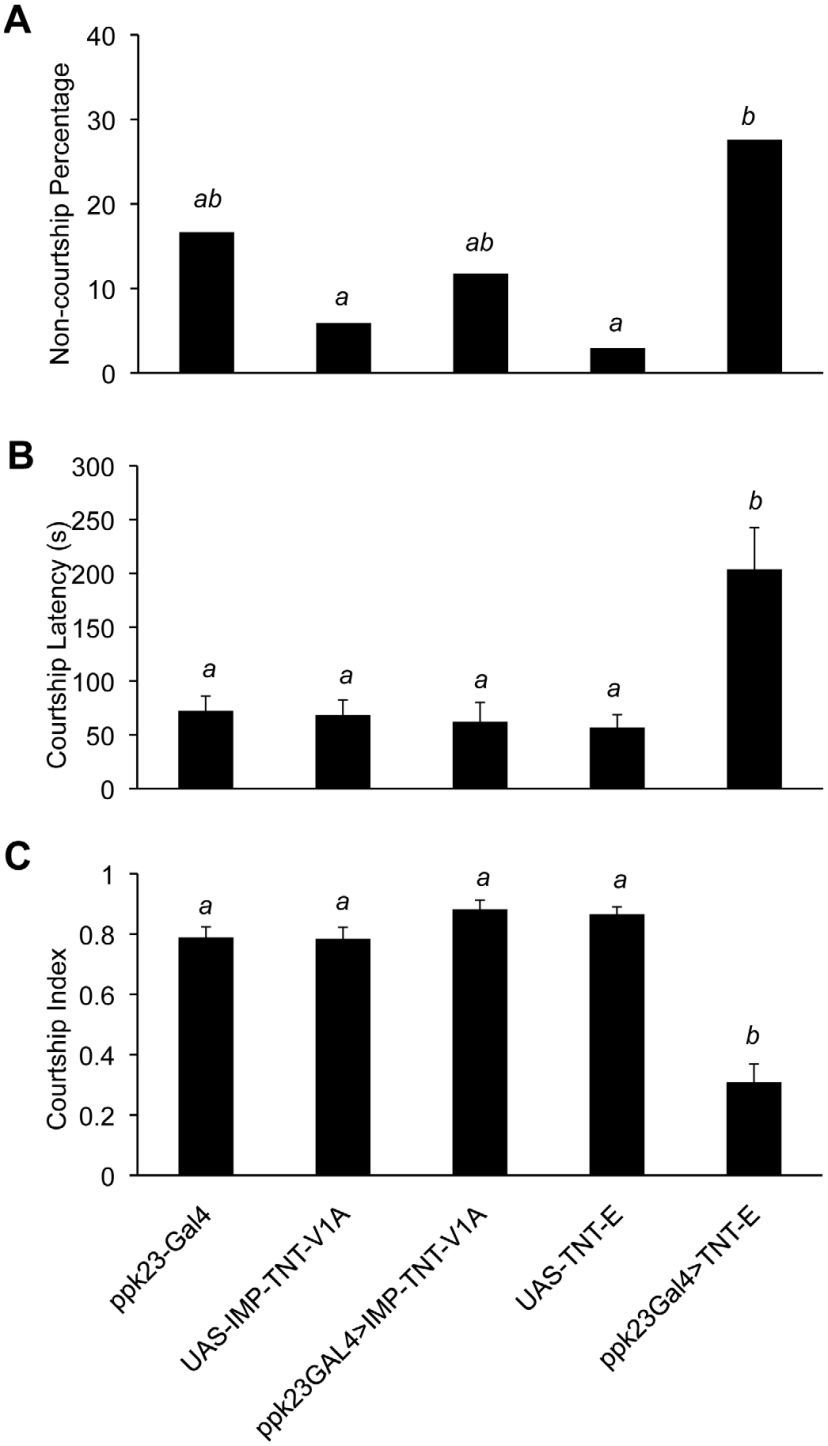
*ppk23*-expressing cells are required for male courtship behavior. (A) Expression of tetanus toxin light chain (TNT) in *ppk23* cells resulted in a high proportion of non-courting males over a 10 min observation time (*Chi-square test*, *p<0.001*). Flies expressing TNT in *ppk23* cells showed increased courtship latency (B; *Kruskal-Wallis rank sum test*, *p<0.001*) and reduced courtship index relative to control crosses (*ppk23*>UAS-IMP-TNT) or parental strains (*ppk23-Gal4*, *UAS-TNT-E* and *UAS-IMP-TNT-V1A* [inactive TNT]) (C; *Kruskal-Wallis rank sum test*, *p<0.001*). n = 29–36 males per each genotype. Error bars denote the standard error of the means. Letters above bars represent the significantly different groups after *post hoc* analyses.

The evidence that *ppk23* expression is sexually dimorphic suggested the hypothesis that *ppk23*-expressing cells play a sensory role in courtship behavior. To test this hypothesis, we blocked neuronal activity in *ppk23*-expressing cells with ectopic expression of TNT using the *UAS-GAL4* system. Inhibiting *ppk23*-expressing neurons increased the proportion of males that failed to demonstrate courtship behavior (Figure 4A). In males that did court, inhibiting *ppk23*-expressing neurons significantly delayed the initiation of courtship when males were exposed to wild-type virgin females (*i.e.*, courtship latency increased, Figure 4B). The courtship index, which is the proportion of time males spent courting in 10 minutes, also fell (Figure 4C).

### *ppk23* contributes to normal male courtship

**Figure 5:**
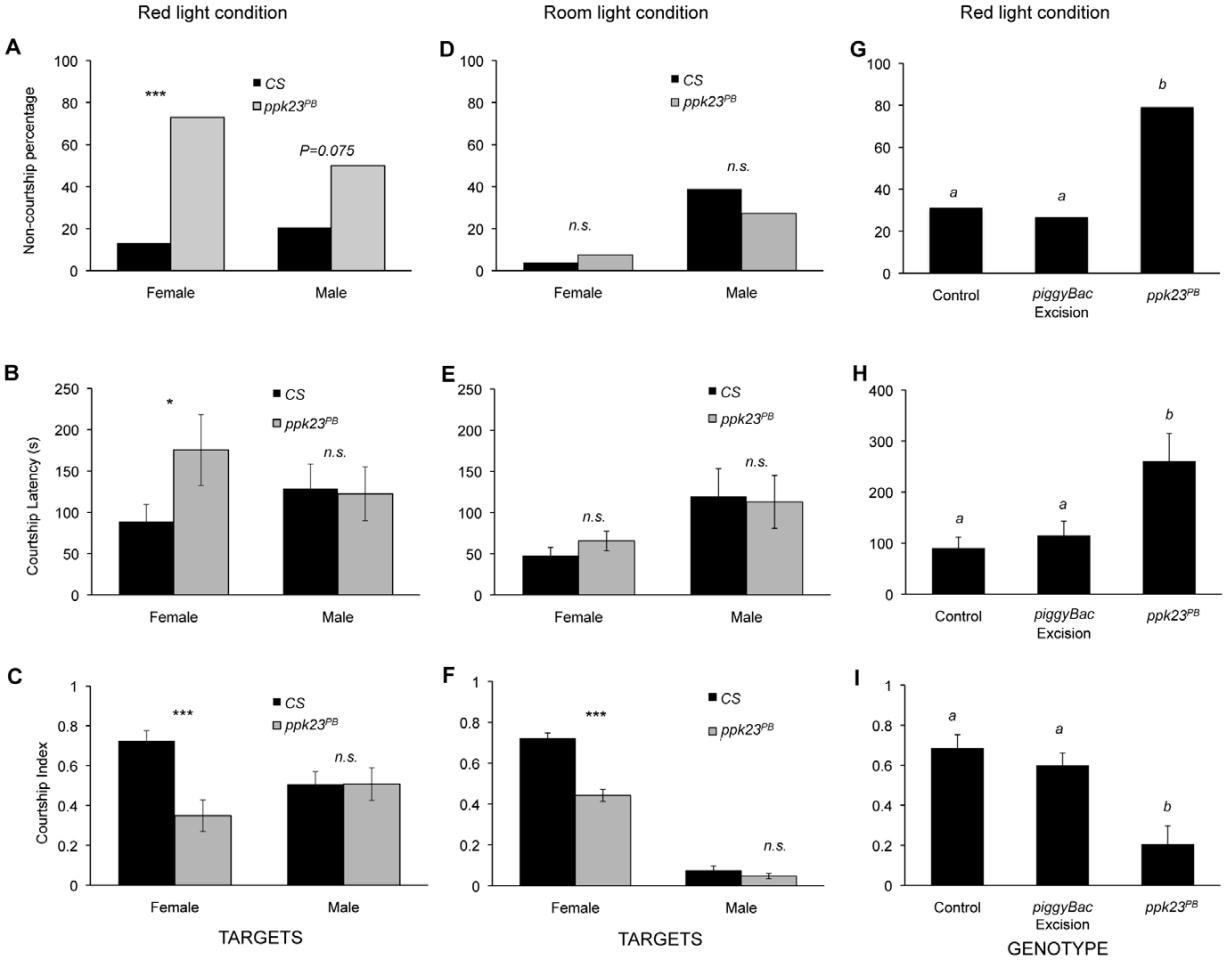
*ppk23* is required for normal male courtship behavior. (A)Significantly higher proportion of *ppk23^PB^* males did not show any courtship behavior in a 10-min observation time under dark (“red light”) conditions towards females (*Chi-square test*; ***, *p<0.001*). (B) *ppk23^PB^* males that did court showed increased courtship latency only towards females (*Two samples wilcoxon test*; *, *p<0.05*) and (C) reduced courtship index (*Two samples wilcoxon test*; ***, *p<0.001*). (D) Under white light conditions, proportion of courting *ppk23^PB^* males was not different than wild types. *Chi-square test*, *N.S.*). (E) No courtship latency differences were detected either (*Two samples wilcoxon test*, *N.S.*). (F) In contrast, *ppk23^PB^* males exhibited reduced courtship index even under white light conditions (*Two samples wilcoxon test*; ***, *p<0.001*). There were no effects of the mutation on any of the measured courtship parameters towards males under white light conditions. (G–I) *piggyBac* excision rescued the effect of *ppk23^PB^* mutation on male courtship behaviors in all measured parameters under red light conditions (G; Chi-square test, *p*<0.001; H; *Kruskal-Wallis rank sum test, p<0.05; I; Kruskal-Wallis rank sum test, p<0.05*). n = 24–38 for each genotype and each condition. *N.S.* indicates no significant difference. Error bars denote the standard errors of the means.

We also asked whether the *ppk23* gene was important for normal courtship by utilizing the insetional hypomorphic allele *ppk23* ^PB^ (Figure S1A). Similarly to the TNT-blocking results, a high proportion of *ppk23^PB^* mutant males did not exhibit obvious courtship behaviors in 10 minutes (Figure 5A). The *ppk23^PB^* mutant males that did court had significantly longer courtship latency and a reduced courtship index towards wild-type mature females (Figure 5B, 5C). The effects of the *ppk23* mutation on male courtship were specific to male-female interactions since we did not observe statistically significant effects of the mutations on naturally occurring male-male courtship interactions (Figure 5A–5C).

We also tested the courtship responses of *ppk23^PB^* mutant flies under normal white (room) light conditions. Under these conditions, more than 90% of the males of both genotypes showed courtship behaviors towards virgin female targets (Figure 5D) without an observable gene effect on courtship latency (Figure 5E). In contrast to latency, *ppk23^PB^* mutant flies still exhibited a reduced courtship index in comparison to wild type controls even under room light conditions (Figure 5F). No effects were observed on male-male courtship under these light conditions (Figure 5D–5F). In further support of the specific role of *ppk23* in courtship behavior, excision of the *ppk23 piggyBac* insertion completely reverted the mutant courtship phenotype to wild type levels for both latency and index (Figure 5G–5I).

**Figure 6:**
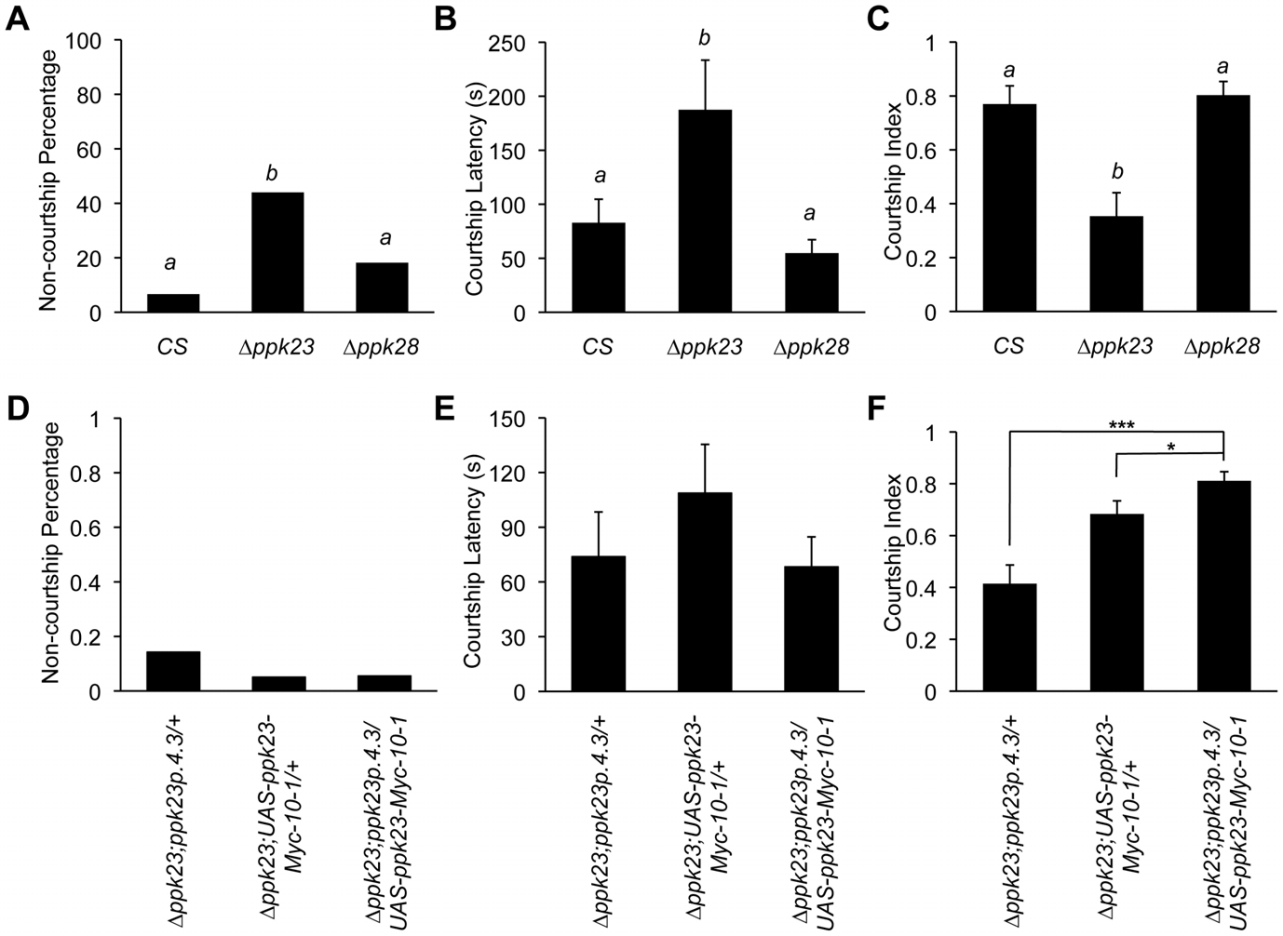
The *ppk23* effect on courtship is not a general property of gustatory DEG/ENaC subunits. (A) Significantly higher proportion of Δ*ppk23* males did not show any courtship behavior in a 10-min observation time under dark (“red light”) conditions towards females (*Chi-square test; p<0.05*). (B) Δ*ppk23* males that did court showed increased courtship latency towards females relatve to CS controls or Δ*ppk28* flies (*Kruskal-Wallis rank sum test, p<0.05*) and (C) reduced courtship index (*Kruskal-Wallis rank sum test*, *p*<0.001). n = 24–27 flies per genotype. Error bars denote the standard error of the mean. Same letters above bars represent groups that were not statistically different in the *post hoc* test. All strains tested were in a common CS genetic background. (D–F) The effect of the Δ*ppk23* allele on courtship index is rescued by overexpression of *ppk23* cDNA (F, ANOVA, *p*<0001, ***; pairwise comparisons using *t*-tests with non-pooled SD, n = 25–30 for each genotype). The effects of the Δ*ppk23* mutations on percent courtship and courtship latency were significantly reduced or absent in the genetic backgrounds of the rescue parental lines relative to flies that carrying Δ*ppk23* alone, which precluded us from observing a significant rescue of these aspects of the *ppk23*-induced courtship phenotype (D and E).

As additional genetic support for the specific role of *ppk23* in male courtship behavior, we utilized recently published complete deletion alleles of *ppk23* (Δ*ppk23*) and *ppk28* (Δ*ppk28*) in courtship assays. These deletion lines were generated independently of the *ppk23^PB^* allele [31]. In agreement with *ppk23^PB^* data, males carrying the null *ppk23* allele (Δ*ppk23* in *CS* background) showed comparable effects with significantly longer courtship latency and reduced courtship index (Figure 6A–6C). In contrast, deletion of the *ppk28* locus, a DEG/ENaC subunit that is enriched in gustatory neurons implicated in water sensing in flies [31], [32], had no impact on male courtship behavior (Figure 6A–6C). Furthermore, overexpressing *ppk23* cDNA in *ppk23*-expressing cells was sufficient to rescue the *ppk23*-induced deficits in courtship index but not courtship latency, which we observed in animals carrying the Δ*ppk23* null allele alone (Figure 6D–6F). Real-time qRT-PCR analyses of *ppk23* mRNA expression in the appendages of Δ*ppk23*; UAS-ppk23cDNA flies detected low basal *ppk23* expression independent of the presence of GAL4 (Figure S2), which may explain the relatively milder phenotype in this background relative to Δ*ppk23* alone. Furthermore, the presence of the mini *white* marker in the transgenic constructs has been recently shown to increase overall sexual activity of males [33], [34], which may have masked some of the effects of the *ppk23* mutation in this background. These data indicate that different DEG/ENaC subunits might contribute to distinct gustatory pathways, some related to feeding and drinking [7], [31], [32], and some to social behaviors.

Mutations in *ppk23* or manipulations of *ppk23*-expressing sensory neurons did not lead to any obvious feeding related phenotypes (Figures S3 and S4). These data further supported the specific role of *ppk23* in mating behaviors. In addition, neither *ppk23^PB^* nor TNT-E-expressing flies showed any obvious developmental or locomotion defects, suggesting the observed courtship phenotype was not due to a general lower activity in males (Figure S5). Furthermore, although males carrying the *PiggyBac* insertion had reduced overall sexual activity, they were not behaviorally sterile. This was supported by the overall viability of the homozygous *ppk23^PB^* and the Δ*ppk23* stocks as well as by directly testing for any obvious behavioral phenotypes in *ppk23^PB^* females, which showed normal sexual receptivity to males (Figure S6).

### *ppk23* plays a role in the response to the female-specific pheromone 7(Z), 11(Z)-Heptacosadiene

**Figure 7:**
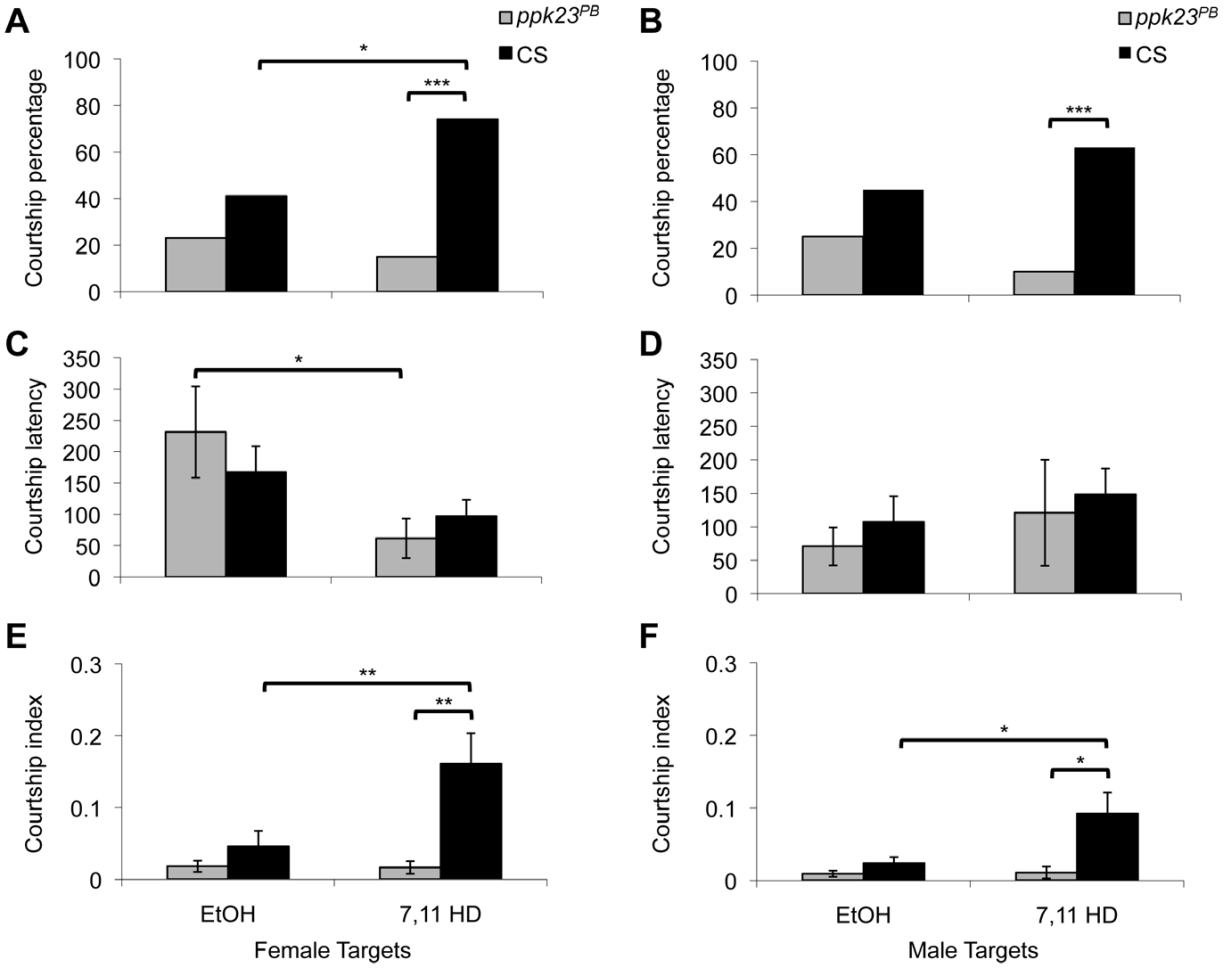
The behavioral response to the aphrodisiac pheromone 7,11-HD is reduced in *ppk23^PB^* flies. (A–B) Larger percent of wild type CS flies exhibited courtship behavior in response to male or female targets laced with 125 ng 7,11-HD (*Chi-squared test*; only significant contrasts are shown; *, *p<0.05*; **, p<0,01; ***, *p*<0.001, n = 20–30 for each genotype per treatment). (C–D) Courtship latency was not affected by the *ppk23* mutation in flies that did court pheromone-laced dummies. (E–F) Significant effects of *ppk23* mutation on courtship index were observed in males that courted pheromone-laced dummies (*Two samples t-test* or *two samples wilcoxon test*, *, *p<0.05*; **, p<0,01; ***, *p*<0.001, n = 20–30 for each genotype per treatment).

Our behavioral and anatomical analyses suggested that *ppk23* might be directly involved in the sensory perception of sex pheromones. To test this hypothesis, we examined the effects of a *ppk23* mutation on the behavioral responses to 7(Z), 11(Z)-Heptacosadiene (7,11-HD), a female specific aphrodisiac pheromone [35]. We first washed the targets flies (male or female CS flies) three times in hexane to remove as much endogenous pheromone as possible. We then applied 7,11-HD or EtOH alone (the solvent for 7,11-HD). Regardless of the sex of the target, wild type males courted pheromone-laced targets significantly more than *ppk23* mutant males as measured by the overall percent courtship (Figure 7A and 7B). We also measured the latency and courtship index of males that initiated courtship of perfumed dummies. As shown in Figure 7C and 7D, there were no significant differences in latency between the two genotypes, which is in agreement with our courtship data of live female targets (Figure 5). In contrast, even when they did initiate courtship, *ppk23* mutant males tended to court the dummies with less intensity than wild type males (Figure 7E and 7F). Although these data do not directly address the issue of whether *ppk23* is directly involved in sensing the pheromone 7,11-HD they do indicate that sensory detection of at least some pheromones requires the function of DEG/ENaC-dependent signaling.

## Discussion

Flies behave in a complex chemical environment that includes signals for both feeding and social interactions. How these different signals are sensed and coded by the nervous system is still poorly understood. Our identification of *ppk23* expression in chemosensory bristles originally suggested that it might contribute to feeding behaviors. However, we detected no gustatory role for *ppk23* or the cells expressing it. Instead, we found that *ppk23* contributes to processes underlying male sexual behavior; *ppk23* was expressed in sexually dimorphic neurons associated with chemosensory bristles in forelegs, and it was required for normal courtship behavior and attraction to the aphrodisiac pheromone 7,11HD in males.

Several lines of evidence suggest that *ppk23* and the cells expressing it are chemosensory. These include the similar morphology of the two neurons that express *ppk23* in each chemosensory bristle, which is in contrast to the single distinctive mechanosensory neuron that is present in each chemosensory bristle [22]. Chemosensory functions are also supported by the defective response of *ppk23* mutant males to the aphrodisiac pheromone 7,11-HD. However, several recent studies also indicated that DEG/ENaC signaling plays a role in mechanosensory functions in the worm and the fly [36], [37]. Therefore, we currently cannot completely exclude the possibility that *ppk23* might also play a role in mechanosensation in the context of mating behavior in flies.

Whether *ppk23* is specifically playing a role in courtship behavior or whether it might be important for other types of social behaviors in the fly is still unknown. In support of a more general role for *ppk23*-dependent signaling in mediating chemically-driven social interactions, a *ppk23* mutation was recently identified in a *p*-elements screen for genes associated with male-male aggression [38]. Together, these data suggest that *ppk23*-dependent signaling might play a general role in social communications, and may affect the response of flies to diverse social-related chemical signals.

Visual cues play an important role in the initiation of courtship behavior in *Drosophila* males, and typically will mask deficits in other sensory modalities [20]. Our data support this, indicating that *ppk23^PB^* males showed normal courtship latencies under normal lighting conditions. These findings suggest that mutations in *ppk23* did not affect the overall sexual drive of males but rather reduced the ability of males to sustain courtship towards female targets in the absence of visual cues, further supporting a chemosensory role for this gene. It also raises an interesting hypothesis; which is that vision (absent under red light condition) is playing a critical role during the initial mate recognition process but is playing a less prominent role in the maintenance of courtship until successful copulation occurs. This is based on our observations that *ppk23^PB^* males showed reduced courtship index under both light conditions but exhibited prolonged courtship latencies under red light but not white light conditions. These results indicate that visual inputs were able to overcome chemosensory deficits during the initial orientation towards a female target but were not enough to overcome such deficits for sustaining courtship. Furthermore, our data also indicate that initial chemo-physical contact between the courting male and the female target represents an important trigger for the release of orientation behavior (which was used to determine courtship latency). This may suggest that PPK23 plays a role in triggering male courtship, which was especially robust under red light conditions in which vision is eliminated.

Although we have established *ppk23* as a gene important for male sexual behaviors, we do not know whether it plays a similar role in females. Our promoter and expression studies suggested that the locus is expressed in appendages of females. It is possible that *ppk23*-expressing cells are playing a role that is relevant to intra-species interactions independent of sex. Our female behavioral data suggested that *ppk23* mutation did not alter female sexual receptivity, but they do not exclude a possible role in other behaviors.

We still do not know whether *ppk23* interacts genetically or physiologically with other receptors previously implicated in *Drosophila* courtship behaviors. Nevertheless, several lines of investigation already suggest that *ppk23* might represent an independent sensory pathway. Typically, gustatory bristles contain either two or four chemosensory neurons, and each expresses a unique set of sensory receptors dedicated to a single taste modality [22]. In contrast, *ppk23* was expressed in a pair of chemosensory neurons innervating a single bristle. Of note, our examination of recently published images of the tarsal expression of *Gr32a*, a chemosensory-related gene involved in inhibiting male-male courtship [39] and the avoidance of aversive chemicals [40], suggests that *Gr32a* is also expressed in pairs of tarsal chemosensory neurons projecting to single sensory bristles. However, In contrast to *ppk23*-expressing neurons, which mostly terminate in the thoracic ganglion, many of the tarsal sensory projections of *Gr32a*-expressing cells form ascending bundles that terminate in the subesophageal ganglion [41], [42]. These data indicate that *ppk23* and *Gr32a* are likely expressed in different sensory neurons, and are playing a role in discrete sensory modalities. This assertion is further supported by previous reports that indicated *Gr32a* is involved in the inhibition of male-male courtship but not in male-female interactions, while we found that *ppk23* plays a role in male-female interactions without any obvious effects on male-male interactions [40], [43]. Other gustatory receptors have also been implicated in courtship behavior. A recent study suggested that *Gr39a* contributes to the male-female courtship and is required in sustaining courtship behavior [44]. These studies suggest a possible relationship between *ppk23* and *Gr39a*, which is currently under investigation. Finally, our data also support a chemosensory role for *ppk7*. Since DEG/ENaC channels often function as heteromultimeric protein complexes[45], [46], these data raise the possibility that *ppk23* and *ppk7* physically interact to form a chemosensory-related channel.

While we do not yet understand the physiological significance of the differences in organization of typical taste-related and *ppk23*-containing bristles, this separation is analogous to the mammalian chemosensory system, which includes dedicated sensory neurons for pheromonal sensing that are independent of the general chemosensory system [47].

Finding *ppk23* expressed in chemosensory neurons and the lack of behavioral responsiveness of *ppk23* males to the aphrodisiac pheromone 7,11-HD suggest that PPK23 is a potential candidate for a pheromone receptor, or for a key component of the pheromonal signaling cascade. In summary, our data indicate that DEG/ENaC signaling contributes to sensory functions underlying sex-related behaviors, and indicate a novel physiological function for this important family of ion channels. Finally, the results presented here further support the idea that insects have evolved separate chemosensory systems for appetitive and pheromonal chemosensory signaling.

## Materials and Methods

### *Drosophila* stocks and cultures

Flies were maintained on standard cornmeal medium at 25°C under 12:12 light-dark cycle. A *ppk23 promoter-GAL4* line was generated by PCR amplifying a 2.6 kb fragment (X: 17402154..17404754), which included the first intron of *ppk23* subcloned into an improved *pPTGAL4* vector [48]. UAS-*ppk23* was generated by cloning the *ppk23* ORF from male appendages into a pUASt vector [49]. Transgenic flies were generated according to standard procedures (Rainbow Genetics Inc. Ca). *UAS-TNT-E* and *UAS-IMP-TNT-V1-A* were obtained from C. O'Kane (Cambridge, England). *UAS-VR1* and the lines expressing three copies of EGFP under direct control of either *Gr5a* or *Gr66a* (*Gr5a>3xEGFP* and *Gr66a*>*3xEGFP*, respectively) were from K. Scott (Berkley, CA). *Gr5a-GAL4* and *Gr66a-GAL4* were from J. Carlson (Yale, CT). The *ppk23^PB^* flies were the *piggyBac* insertion allele of *ppk23* (line c03836, Harvard Exelixis collection), which were outcrossed to the Canton-S (CS) background for six generations. We also used *piggyBac* transposase to remove the *piggyBac* insertion in the *ppk23* locus according to standard genetic method [50]. The Δ*ppk23* and Δ*ppk28* alleles were from the Wang lab [31]. The original published genetic background that carried these alleles had highly reduced overall male courtship behavior likely due to background mutations. Hence, both published alleles were outcrossed into the wild type CS background for six generations prior to the described behavioral studies. Unless mentioned, other fly strains used in our studies were obtained from the Bloomington Stock Center.

### RNA analysis

Flies were separated by sex under CO_2_ and kept at −80°C until processing. To separate body parts, microcentrifuge tubes with flies were dipped in liquid nitrogen and then separated by repeated vortexing. Total RNA from tissues was extracted with the Rneasy mini kit (Qiagen) or Trizol reagent (Invitrogen) according to manufacturer instructions. RT-PCR analysis was performed by using the SuperScript II reverse transcriptase (Invitrogen) with 1 µg total RNA in 20 µl reaction according to manufacturer instructions. PCRs were performed with the ACCUprime *pfx* supermix (Invitrogen) in 25 µl reactions, and were subsequently separated on a 1.2% agarose gel. Real-time quantitative RT-PCR assays for *ppk23* were performed on an “ABI7500 fast” machine with an ABI predesigned probe-based assay (*ppk23*; Dm01839671_g1) according to manufacturer instructions. For all other assays, gene specific primers were designed with the PrimerExpress package (ABI Inc.) and were analyzed using Power SYBR kit (ABI Inc.). See Table S1 for primer sequences. The housekeeping gene *rp49* were used as an RNA loading control (Dm02151827_g1). Data were transformed according to the ΔΔCt method and are represented as relative values [51]. Northern blot analysis was done with the NorthernMax kit (Ambion), using a DIG-labeled *ppk23* specific cRNA probe (Roche). The RNA probe was generated via *in vitro* transcription using a PCR template that included nucleotides 578–1369 of *ppk23*-RA. The probe was designed to hybridize to all three possible alternatively spliced *ppk23* transcripts.

### Feeding behavior assay

Groups of 20–30 four to seven day-old flies of mixed sexes were kept overnight in an empty fly vial containing a wet paper towel. On test day, flies were briefly immobilized on ice and then transferred to the test plate. Test plates were 60-well Nunc dishes, which had alternate wells filled with 10 µl of either test compound mixed with 2 mM sucrose or 2 mM sucrose alone (a weak feeding inducer) in 1% agarose. Food dye (1% v/v) was added to each alternative choice (either red or blue). Flies were allowed to feed for 2 h in the dark and were then frozen in a −20°C freezer. Only flies that showed clear dye in their digestive system were further used for index calculation. Choice index was calculated as (# of flies that consumed tested compound + ½ purple [consumed both])/total # of flies that had any dye in their abdomen. Choice index of 0.5 indicated that the consumption of the tested compound was not different than 2 mM sucrose. Any deviation towards index of 1 or 0 reflected a positive or negative preference, respectively of the compound relative to 2 mM sucrose. Concentrations for various compounds are in figure legends, except for in Figure 3D; yeast was 0.16% v/v live Baker's yeast paste. *E. coli* (DH5α strain) was 10% v/v overnight culture in standard LB medium. “Old food” was 10% v/v one-week-old standard *Drosophila* culture food from a high-density population. Monosodium glutamate (MSG) was 30 mM. Bovine serum albumin (BSA) was 2% w/v. No obvious differences between males and females were observed in our assays. Consequently, data were collected from mixed sex assays.

### Proboscis extension response

Proboscis extension was assayed in 2 day old males as previously described [17] with the following modifications. 1 d old flies were starved for approximately 24 hours, then immobilized by chilling on ice and mounted ventral-side-up using myristic acid. Flies were allowed to recover for two hours in humid conditions. To begin the experiment, flies were satiated with water until no proboscis extension was elicited by water stimulation. Next, flies were tested by touching a drop of a solution on a pipette tip to a foreleg and scored for full proboscis extension. Flies were given three trials of same substance in each concentration with water application in-between trials; only flies that responded at least 2 out of 3 times were considered ‘responders’ for that concentration. For bitter assays, 100 mM sucrose was included at all concentrations of caffeine. At the beginning and the end of each assay, flies were tested for response to 100 mM sucrose and any fly failing to respond was excluded from the study. The “percent responders” for each genotype at each concentration was calculated by tallying the number of flies responding divided by the total number of flies tested. The responding index represents the cumulative scores (sum of all responses of an individual animal to a specific sequence of tarsal stimuli).

### Courtship behavior

Courtship behavior was assayed with four to seven day old males as previously described [17] with the following modifications. Newly emerged males were kept in individual vials with food until tested. Courtship assays were done under the red light conditions unless differently stated and the targets were the decapitated flies. Courtship latency was calculated as the time from female introduction until the male showed obvious courtship behavior such as orientation coupled with wing extensions. In wild type males, first orientation was typically concurrent with first contact with a female. In all cases when we observed a delayed latency, the initiation of courtship required multiple contacts between males and females. Males were observed for 10 min. Once courtship began, courtship index was calculated as the proportion of time a male spent in any courtship-related activity during a 10 min period or until mating occurred. For the 7,11-HD experiments, all the targets used were virgin 5 days old flies and freshly cleaned the cuticle hydrocarbon by 3 times 5-min hexane washes, then were applied 2.5 ul of 50 ng/ul 7,11-HD or the control solvent, 100% ethanol. All assays were performed under normal light conditions in circular courtship arenas, 22 mm in diameter. Tests for general locomotion were performed as previously described [52], [53].

### Statistical procedures

All statistical tests were performed using the R statistical package. Data were tested for normality by using the *Shapiro-Wilk* test. Two-sample *t*-tests and one-way *ANOVA* tests were used for parametric statistics and the two-samples *wilcoxon* test and *Kruskal-Wallis* rank sum test were used for non-parametric tests. *Chi-square* tests were used for examining frequency-based data.

## Supporting Information

Figure S1 The *ppk23 locus*. (A) Line c03836 from the Harvard Exelixis collection is an insertion of a *piggyBac* transposon in the *ppk23* locus (*ppk23^PB^*). The *piggyBac* insertion was verified by plasmid rescue and was found to be in the second intron, shared by all predicted *ppk23* transcripts. *ppk23*-RX represents the main transcript we have identified in appendages, which does not fully correspond to the currently predicted transcripts (RA-RC). Black boxes represent coding exons. Gray boxes represent untranslated regions. Red arrows represent the location of the primers used in real-time qRT-PCR analyses of *ppk23*. Orange boxes represent the probe used for the northern blot analyses. (B) Real-time quantitative RT-PCR analysis reveals that *ppk23* expression is enriched in the appendages in males and females. No expression was detected in heads or bodies. Data are relative mRNA fold differences (n = 4 per group). (C) Real-time quantitative RT-PCR analysis indicates that *ppk23^PB^* flies have reduced levels of *ppk23* transcripts in male appendages (n = 4, *t*-test, *p*<0.01), indicating the allele is likely a hypomorph. Data are relative mRNA fold difference in wild-type relative to *ppk23^PB^* flies. (D) *ppk23* expression pattern in each legs (F: foreleg; M: middle leg; H: hind leg. Genotype: *ppk23*-Gal4>UAS-nls-GFP).
(TIF)

Figure S2 Real-time quantitative RT-PCR analysis indicates that Δ*ppk23*; UAS-*ppk23*cDNA flies express low levels of *ppk23* transcripts independent of the presence of GAL4. Data are relative mRNA fold differences.
(TIF)

Figure S3 Taste responses of *ppk23* ^PB^ flies to various tastants. Behavior of adult flies in taste choice assay; choice index is described in Methods section. (A) Response to indicated concentrations of the appetitive sugar trehalose. (B) Response to indicated bitter compounds. Unless noted, all compounds were used at 10 mM. (C) Response to high and low concentrations of salts. (D) Response to a variety of stimuli that either induce or repress feeding response. There were no differences in taste choice behavior between wild type controls and the *ppk23^PB^* flies in any of the tests (*t*-test, N.S.; n = 3–10 trials per group across all experiments). (E–G) *ppk23^PB^* flies showed normal proboscis extension reflex in response to the different concentration of sucrose (E–F) and caffeine (G–H) when compared with the CS wild-type controls. (Chi-squared test for the proportion comparison, *t*-test for the responding index, N.S., n = 30 per genotype). Error bars denote standard errors of the mean.
(TIF)

Figure S4 *ppk23* chemosensory receptor neurons do not contribute to appetitive behaviors. (A) Behavior of adult flies in taste choice assay; choice index is described in Methods section. Controls (Cont) were *ppk23-GAL4 x UAS-TNT^inactive^* flies and experimental flies were of *ppk23-GAL4 x UAS-TNT-E* (TNT). Compounds were at the following concentration: trehalose, 50 mM; caffeine, 10 mM; KCl, 100 mM; citric acid, 1 M; monosodium glutamate (MSG), 30 mM. Twenty to thirty flies were used in each trial per test plate. Three to ten trials per compound were analyzed. (B) Z-stack confocal image of last tarsal segment from fly carrying *ppk23-GAL4 x UAS-DsRed* (red soma, solid arrows) and *EGFP* directly driven by the *Gr5a* promoter (*Gr5a>3xEGFP*; green soma, dashed arrows). (C) Z-stack confocal image of last tarsal segment from fly carrying *ppk23-GAL4 x UAS-DsRed* (red soma, solid arrows) and *EGFP* directly driven by the *Gr66a* promoter (*Gr66a>3xEGFP*; green soma, dashed arrows). In both (B) and (C), green and red cells do not project to the same sensory bristles. (D) Behavioral response to capsaicin. All *promoterX-GAL4* lines were crossed to *UAS-VR1* flies. *Gr66a-GAL4* (bitter receptor neurons) and *Gr5a-GAL4* (sweet receptor neurons) were positive controls for repulsion or attraction to capsaicin, respectively. *w* ^1118^ controls were the same background used for producing *ppk23-GAL4* transgenic flies and were used for the wild-type control cross. Capsaicin test plates were 2 mM sucrose versus 2 mM sucrose+0.01 mM capsaicin [56]. *, *p*<0.05 (N = 4–7 groups of 20–30 flies per genotype). (E–H) Proboscis extension reflex responses of flies after blocking *ppk23*-expressing cells with tetanus toxin (TNT). Flies expressing the inactive form of TNT (IMP-TNT) were used as wild type control. (E–F) Induction of the proboscis extension reflex in response to increasing concentrations of sucrose. One parental line, *ppk23*-Gal4, showed higher sensitivity to the low concentration of sucrose (E, Chi-squared test, *p*<0.01, **, [sucrose] = 1 mM; *p*<0.05, *, [sucrose] = 5 mM) and high responding index relative to the other parental genotypes (ANOVA, *p*<0.01, **). Nevertheless, there were no differences in sucrose responsiveness between the two experimental groups that either expressed the active or inactive forms of TNT in *ppk23*-expressing neurons (n = 30 for each genotype per treatment). (G–H) Inhibition of the proboscis extension reflex in response to increasing concentrations of the bitter chemical caffeine. (G) Overall, inhibition of *ppk23*-expressing neurons did not alter the responsiveness to caffeine except for a mild effect at the 10 mM concentration (Chi-squared test, p = 0.05 for total 5 groups, *p*<0.05 for ppk23>TNT-E flies and ppk23-Gal4/UAS-TNT-E flies, n = 30 for each genotype per treatment). No differences were found in the overall caffeine responding index (ANOVA, N.S.). Error bars denote standard error of the means.
(TIF)

Figure S5 *ppk23* mutation or TNT expression in *ppk23* cells have no effect on general locomotion. (A) Climbing score of *ppk23* ^PB^ flies and matched CS wild type control (see methods). There was no effect of the *ppk23* mutation on male climbing (*t*-test, n = 10 groups, 10 flies per group). (B) Walking score of *ppk23* ^PB^ flies and CS wild type flies. The number of times that a male flies crossed the bisecting line of the test chamber in a minute was recorded. There was no effect of the *ppk23* mutation on male general locomotion (*t*-test, n = 12 flies per genotype). (C) There was no effect of TNT expression in *ppk23* cells on male climbing. All experimental and controls genotypes are as in Figure 4. (D) There was no effect of the TNT expression in *ppk23* cells on male general locomotion. (*Kruskal-Wallis rank sum test, p<0.001*, n = 12–15 flies per genotype). N.S. indicates no significant difference. Error bars denote the standard error of the mean.
(TIF)

Figure S6 *ppk23* mutation has no effect on male copulation success or female sexual receptivity. Courtship responses of CS wild type males to *ppk23* ^PB^ and CS wild type females. (A) There were no effects of the *ppk23* mutation in target females on courtship index of CS wild type males flies (*t*-test, n = 15 per target genotype). (B) There was no effect of ppk23 mutations on female receptivity measured by her copulatory success. (*Chi-square test*, N.S. indicates no significant difference. Error bars denote the standard error of the means.
(TIF)

Table S1 List of real-time q-RT-PCR primers.
(DOC)
