# Supplementary figures and images for: ppk23-Dependent Chemosensory Functions Contribute to Courtship Behavior in Drosophila melanogaster
Source: PLoS Genet. 2012 Mar 15;8(3):e1002587. doi: 10.1371/journal.pgen.1002587 (PMC3305452; doi:10.1371/journal.pgen.1002587)

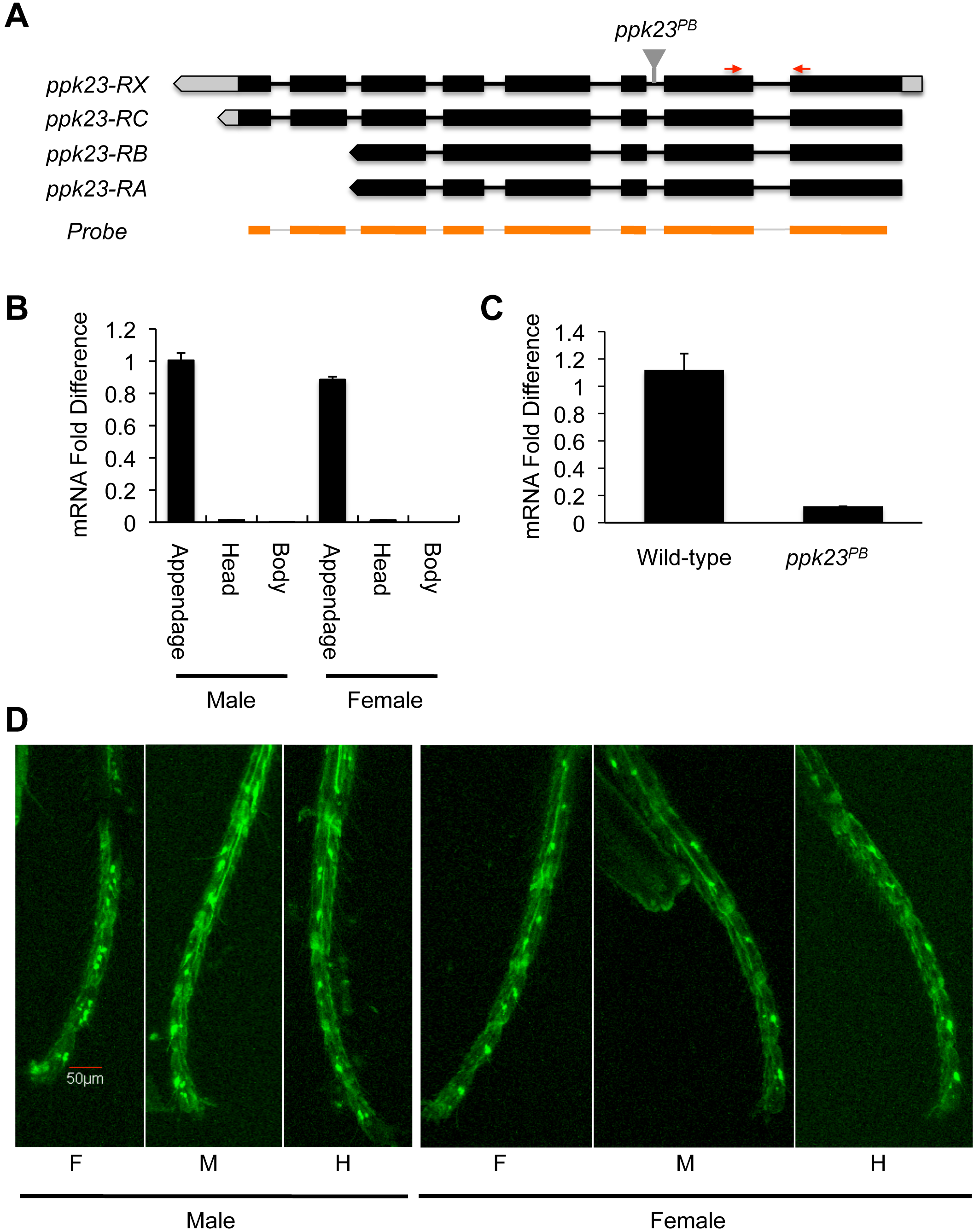

Supplement: Figure S1 — The ppk23 locus. (A) Line c03836 from the Harvard Exelixis collection is an insertion of a piggyBac transposon in the ppk23 locus (ppk23PB). The piggyBac insertion was verified by plasmid rescue and was found to be in the second intron, shared by all predicted ppk23 transcripts. ppk23-RX represents the main transcript we have identified in appendages, which does not fully correspond to the currently predicted transcripts (RA-RC). Black boxes represent coding exons. Gray boxes represent untranslated regions. Red arrows represent the location of the primers used in real-time qRT-PCR analyses of ppk23. Orange boxes represent the probe used for the northern blot analyses. (B) Real-time quantitative RT-PCR analysis reveals that ppk23 expression is enriched in the appendages in males and females. No expression was detected in heads or bodies. Data are relative mRNA fold differences (n = 4 per group). (C) Real-time quantitative RT-PCR analysis indicates that ppk23PB flies have reduced levels of ppk23 transcripts in male appendages (n = 4, t-test, p<0.01), indicating the allele is likely a hypomorph. Data are relative mRNA fold difference in wild-type relative to ppk23PB flies. (D) ppk23 expression pattern in each legs (F: foreleg; M: middle leg; H: hind leg. Genotype: ppk23-Gal4>UAS-nls-GFP). (TIF) [file pgen.1002587.s001.tif]

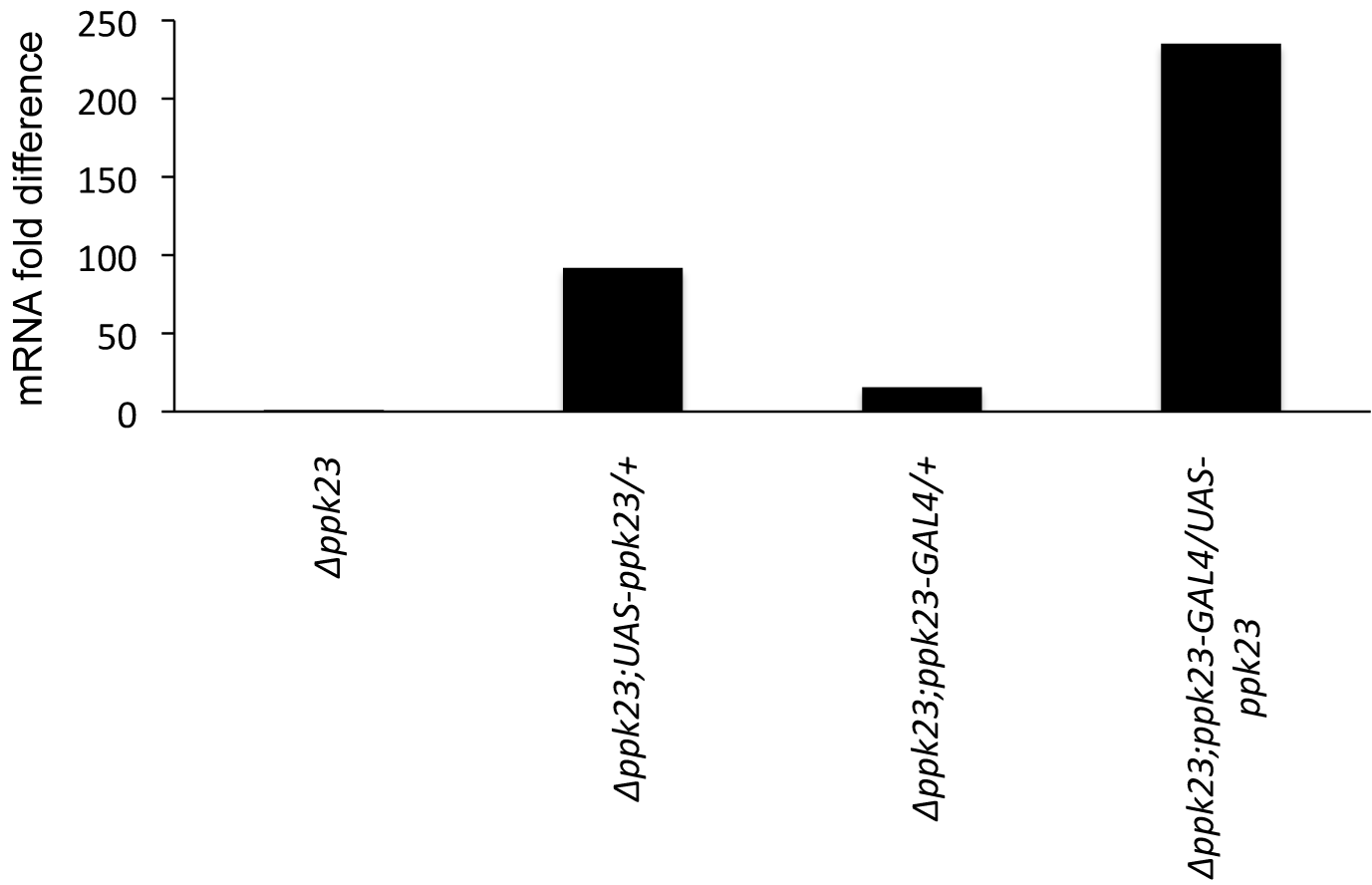

Supplement: Figure S2 — Real-time quantitative RT-PCR analysis indicates that Δppk23; UAS-ppk23cDNA flies express low levels of ppk23 transcripts independent of the presence of GAL4. Data are relative mRNA fold differences. (TIF) [file pgen.1002587.s002.tif]

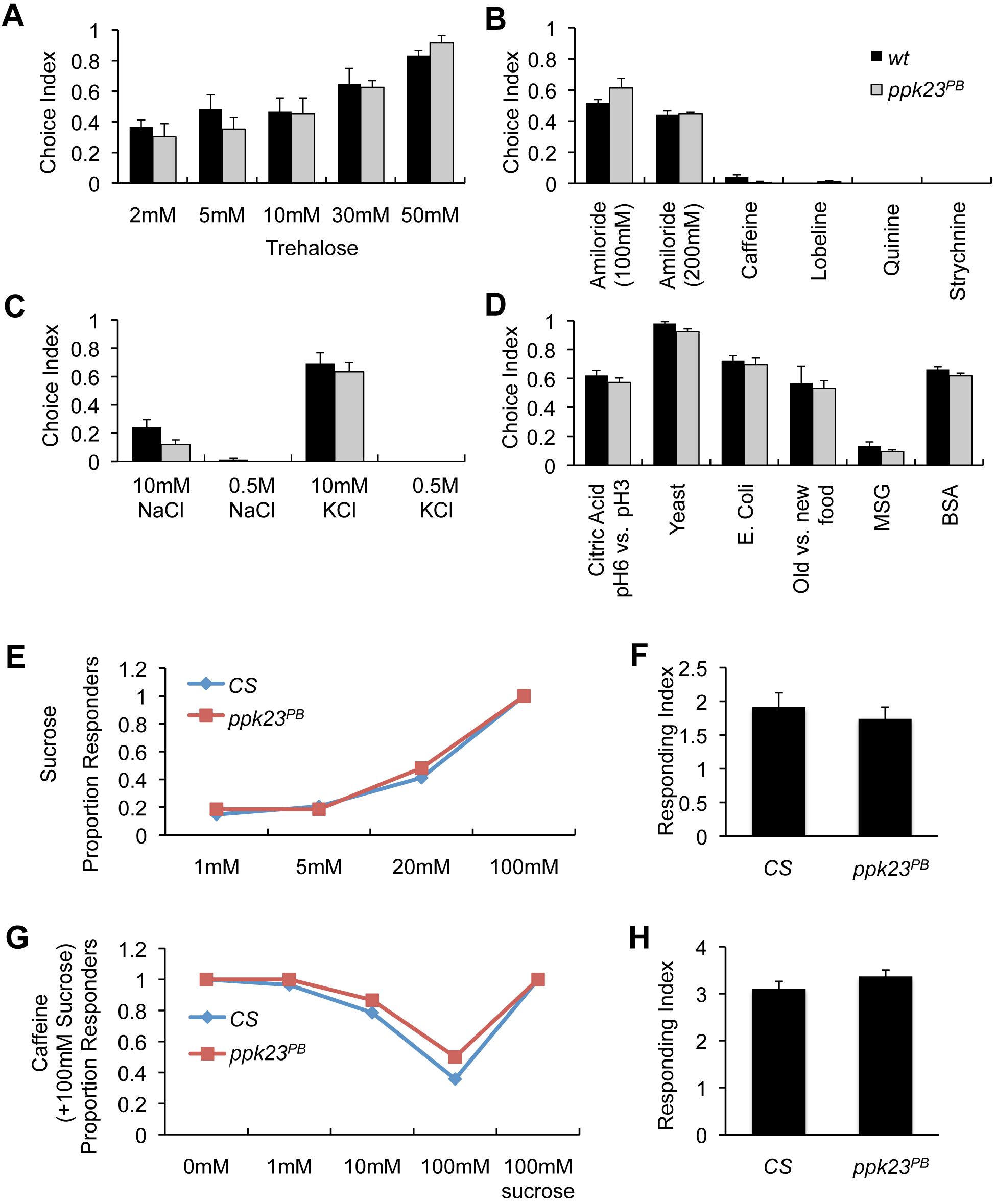

Supplement: Figure S3 — Taste responses of ppk23 PB flies to various tastants. Behavior of adult flies in taste choice assay; choice index is described in Methods section. (A) Response to indicated concentrations of the appetitive sugar trehalose. (B) Response to indicated bitter compounds. Unless noted, all compounds were used at 10 mM. (C) Response to high and low concentrations of salts. (D) Response to a variety of stimuli that either induce or repress feeding response. There were no differences in taste choice behavior between wild type controls and the ppk23PB flies in any of the tests (t-test, N.S.; n = 3–10 trials per group across all experiments). (E–G) ppk23PB flies showed normal proboscis extension reflex in response to the different concentration of sucrose (E–F) and caffeine (G–H) when compared with the CS wild-type controls. (Chi-squared test for the proportion comparison, t-test for the responding index, N.S., n = 30 per genotype). Error bars denote standard errors of the mean. (TIF) [file pgen.1002587.s003.tif]

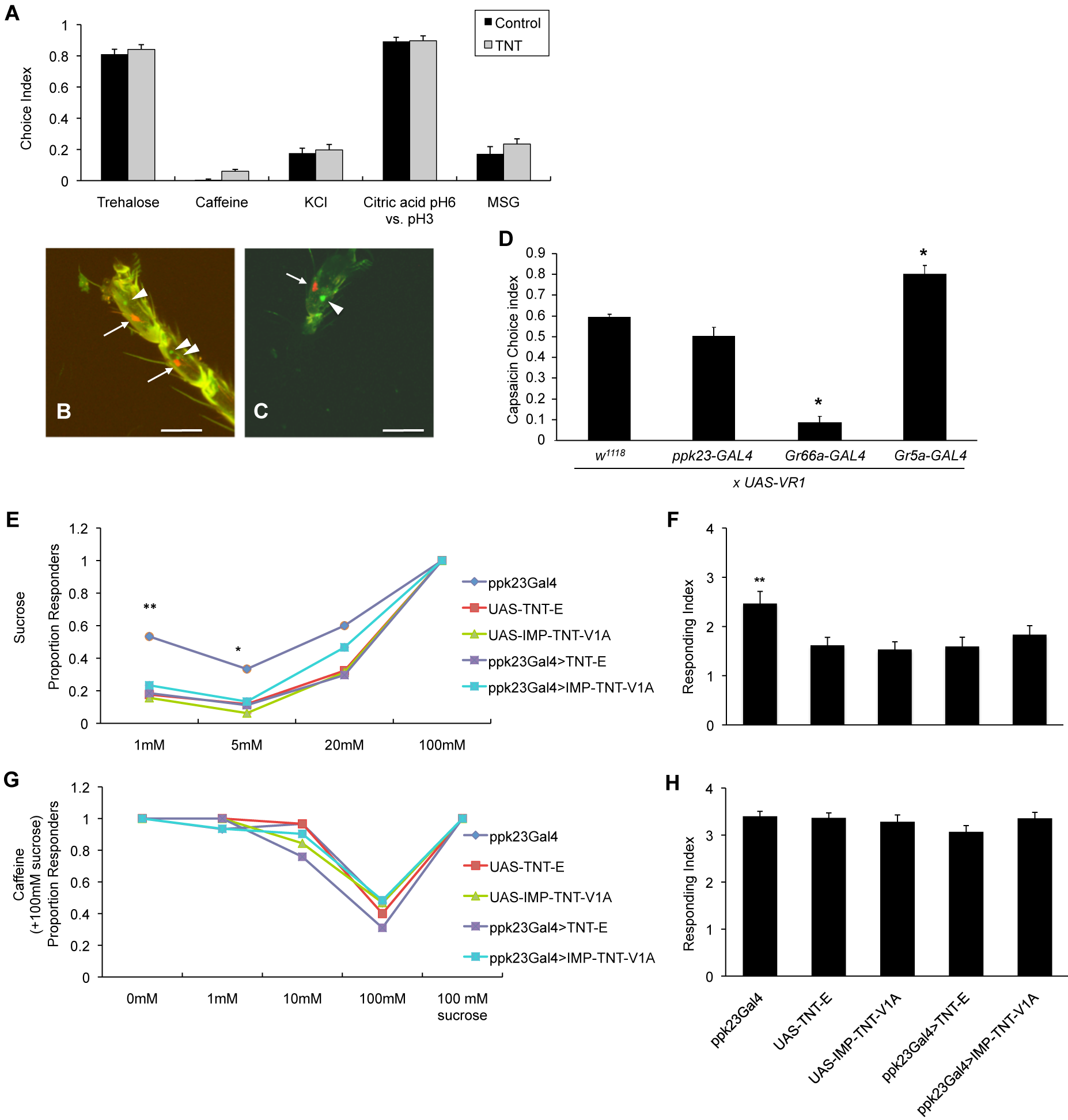

Supplement: Figure S4 — ppk23 chemosensory receptor neurons do not contribute to appetitive behaviors. (A) Behavior of adult flies in taste choice assay; choice index is described in Methods section. Controls (Cont) were ppk23-GAL4 x UAS-TNTinactive flies and experimental flies were of ppk23-GAL4 x UAS-TNT-E (TNT). Compounds were at the following concentration: trehalose, 50 mM; caffeine, 10 mM; KCl, 100 mM; citric acid, 1 M; monosodium glutamate (MSG), 30 mM. Twenty to thirty flies were used in each trial per test plate. Three to ten trials per compound were analyzed. (B) Z-stack confocal image of last tarsal segment from fly carrying ppk23-GAL4 x UAS-DsRed (red soma, solid arrows) and EGFP directly driven by the Gr5a promoter (Gr5a>3xEGFP; green soma, dashed arrows). (C) Z-stack confocal image of last tarsal segment from fly carrying ppk23-GAL4 x UAS-DsRed (red soma, solid arrows) and EGFP directly driven by the Gr66a promoter (Gr66a>3xEGFP; green soma, dashed arrows). In both (B) and (C), green and red cells do not project to the same sensory bristles. (D) Behavioral response to capsaicin. All promoterX-GAL4 lines were crossed to UAS-VR1 flies. Gr66a-GAL4 (bitter receptor neurons) and Gr5a-GAL4 (sweet receptor neurons) were positive controls for repulsion or attraction to capsaicin, respectively. w 1118 controls were the same background used for producing ppk23-GAL4 transgenic flies and were used for the wild-type control cross. Capsaicin test plates were 2 mM sucrose versus 2 mM sucrose+0.01 mM capsaicin [56]. *, p<0.05 (N = 4–7 groups of 20–30 flies per genotype). (E–H) Proboscis extension reflex responses of flies after blocking ppk23-expressing cells with tetanus toxin (TNT). Flies expressing the inactive form of TNT (IMP-TNT) were used as wild type control. (E–F) Induction of the proboscis extension reflex in response to increasing concentrations of sucrose. One parental line, ppk23-Gal4, showed higher sensitivity to the low concentration of sucrose (E, Chi-squared test, p<0.01, ** [file pgen.1002587.s004.tif]

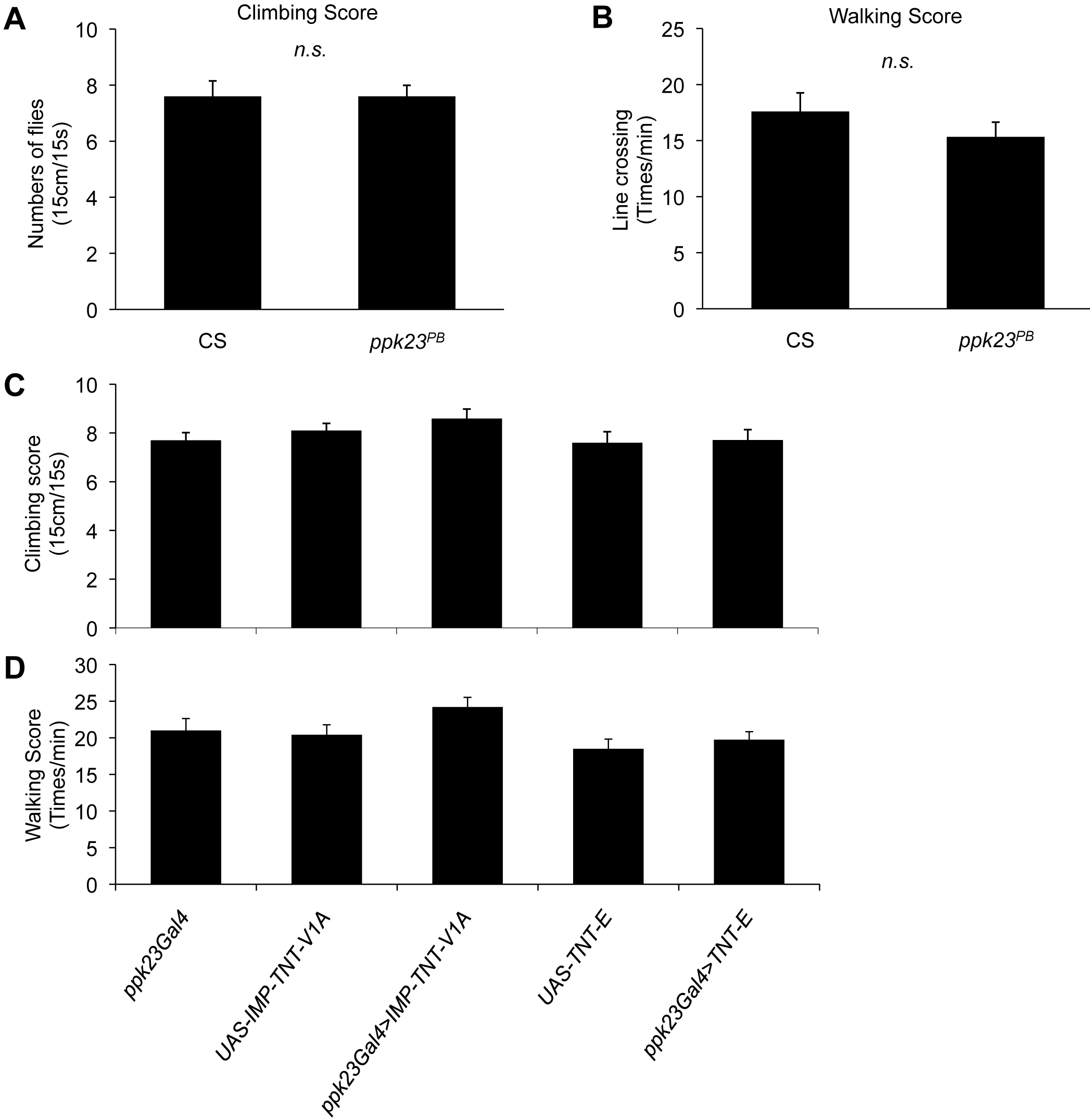

Supplement: Figure S5 — ppk23 mutation or TNT expression in ppk23 cells have no effect on general locomotion. (A) Climbing score of ppk23 PB flies and matched CS wild type control (see methods). There was no effect of the ppk23 mutation on male climbing (t-test, n = 10 groups, 10 flies per group). (B) Walking score of ppk23 PB flies and CS wild type flies. The number of times that a male flies crossed the bisecting line of the test chamber in a minute was recorded. There was no effect of the ppk23 mutation on male general locomotion (t-test, n = 12 flies per genotype). (C) There was no effect of TNT expression in ppk23 cells on male climbing. All experimental and controls genotypes are as in Figure 4. (D) There was no effect of the TNT expression in ppk23 cells on male general locomotion. (Kruskal-Wallis rank sum test, p<0.001, n = 12–15 flies per genotype). N.S. indicates no significant difference. Error bars denote the standard error of the mean. (TIF) [file pgen.1002587.s005.tif]

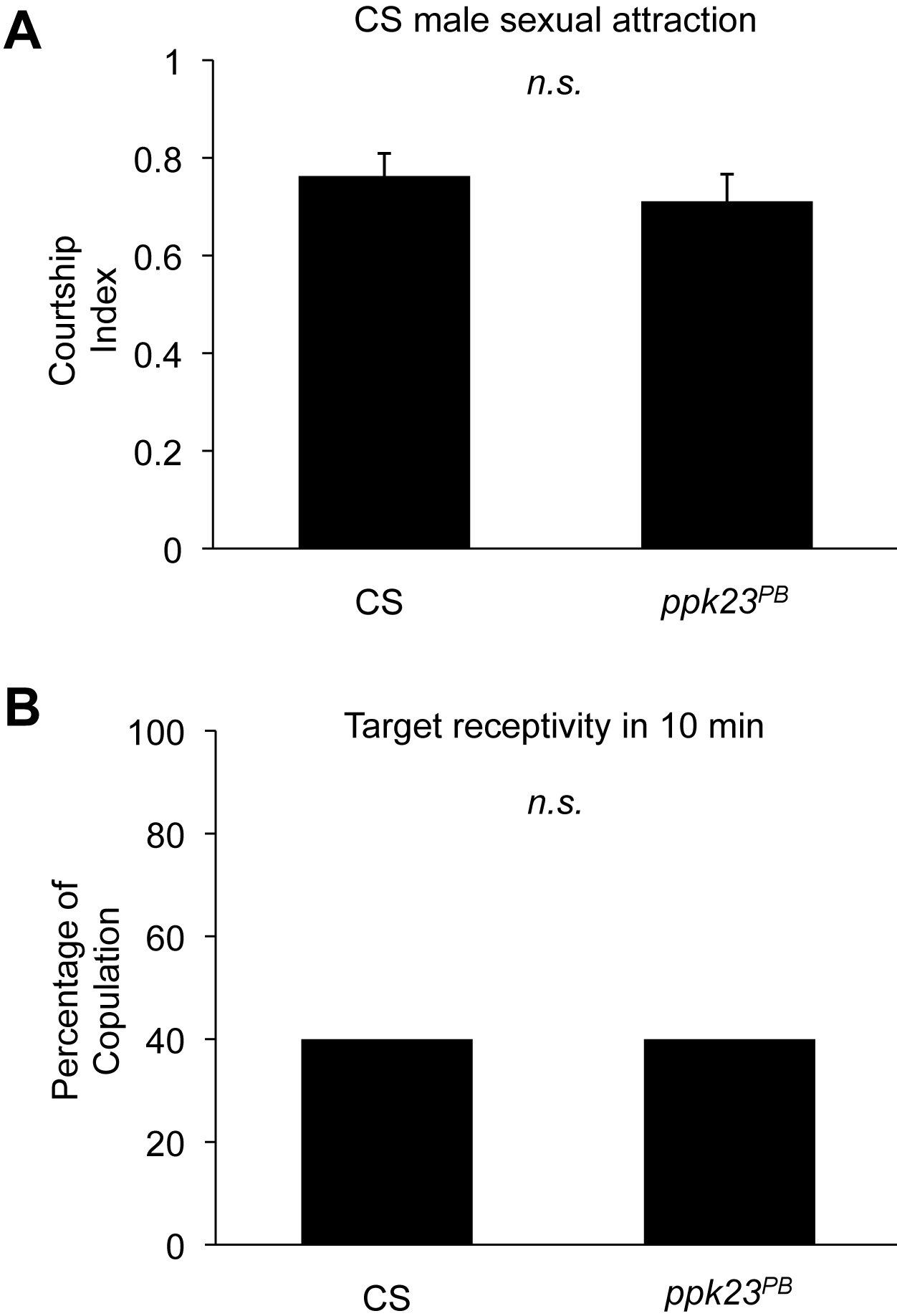

Supplement: Figure S6 — ppk23 mutation has no effect on male copulation success or female sexual receptivity. Courtship responses of CS wild type males to ppk23 PB and CS wild type females. (A) There were no effects of the ppk23 mutation in target females on courtship index of CS wild type males flies (t-test, n = 15 per target genotype). (B) There was no effect of ppk23 mutations on female receptivity measured by her copulatory success. (Chi-square test, N.S. indicates no significant difference. Error bars denote the standard error of the means. (TIF) [file pgen.1002587.s006.tif]
