# Supplementary material for: ppk23-Dependent Chemosensory Functions Contribute to Courtship Behavior in Drosophila melanogaster
Source: PLoS Genet. 2012 Mar 15;8(3):e1002587. doi: 10.1371/journal.pgen.1002587 (PMC3305452; doi:10.1371/journal.pgen.1002587)
Supplement: Table S1 — List of real-time q-RT-PCR primers. (DOC) [file pgen.1002587.s007.doc]

**Table S1**

Real-Time q-RT-PCR primers sequences

| Gene name | Forward primer | Reverse primer |
| --- | --- | --- |
| *rpk* | ACAGCCAGTTCAGCGAAGAT | CACTAACGTTTTCGGGCCTA |
| *ppk3* | TGGACGTGTTCACCCTGTTA | CCCTCATGTGGTCGTCTCTT |
| *ppk4* | TACGCTGGAATTGTGACGAA | GACCGAAAAACGTCTTGGAA |
| *ppk6* | GCTCCTGCTGAGCATCTACC | AGTTGACCACCTTCGGACTG |
| *ppk7* | CTGGCCTATTTCCTGTGCAT | GGCGTGGTTTGTTGAAAGTT |
| *ppk13* | GGTCTGGTCTGTGATTGCCT | TATGAGGCCCACTTCGATTC |
| *ppk16* | GGCAGTAGTTGCGCATGTTCT | CCAGCCGCAACATCGATT |
| *ppk19* | GAATCAACGATGCACAATGC | TTGTCTGGGCAATCACAAAA |
| *ppk20* | TCGCGGATTTGTTAAAGGTC | GGTGGACTTCTCGCAGTAGC |
| *ppk21* | TCTGCTGACCACCAGCATAG | GCGCACCGTTTGATAAAGTT |
| *ppk23* | ACCGACTTCCACAACCA | GGGTATACATTTGGGCCTG |
| *ppk24* | ATGCCAGCCATTTCTACGAC | GCGAAGGGCATTGTCTATGT |
| *llz (ppk25)* | CATATCGCAAGTGTCGCT | CACTCAAGTCGGCAAAGA |
| *ppk28* | ATGAGGACGTTGACGGAAAG | CGCCCTGCTACAGATGGTAT |
| *ppk29* | CGCATAGATCCGTCGAAAAT | GCGAACCAGCGAAGTTTTAG |
| *nan* | CGTCACCGTTGTGATAATGC | GAAGGGACCGACAGTTTTGA |
| *nompC* | GATCCTTCAAGCCACCGATA | GTGTGGGCAATCACTTCCTT |
| *pain* | CGAGCCTTCGATGTCATTTT | CCTTAAGCAGTCGCTCCAAC |
| *TrpA1* | TGCTGTACATCTCGGCTTTG | TGCAGGAACAGTAGCAATCG |
| *CheB42a* | GACGAGATTAAGGAAAAGTGCT | TCAAGTCCAGTTCGTAGTCT |
| *rp49* | CACCAAGCACTTCATCCG | TCGATCCGTAACCGATGT |
